# Expression of *FOXL2* and *RSPO1* in Hen Ovarian Follicles and Implication of Exogenous Leptin in Modulating Their mRNA Expression in In Vitro Cultured Granulosa Cells

**DOI:** 10.3390/ani9121083

**Published:** 2019-12-04

**Authors:** Weihe Niu, Izhar Hyder Qazi, Sichen Li, Xiaoling Zhao, Huadong Yin, Yan Wang, Qing Zhu, Hongbing Han, Guangbin Zhou, Xiaohui Du

**Affiliations:** 1 Farm Animal Genetic Resources Exploration and Innovation Key Laboratory of Sichuan Province, College of Animal Science and Technology, Sichuan Agricultural University, Chengdu 611130, China; weiheniusiacu@163.com (W.N.); vetdr_izhar@yahoo.com (I.H.Q.); cdlisichen@sina.com (S.L.); zhaoxiaoling@sicau.edu.cn (X.Z.); yinhuadong@sicau.edu.cn (H.Y.); as519723614@163.com (Y.W.); zhuqingsicau@163.com (Q.Z.); 2 Department of Veterinary Anatomy and Histology, Shaheed Benazir Bhutto University of Veterinary and Animal Sciences, Sakrand 67210, Sindh, Pakistan; 3 National Engineering Laboratory for Animal Breeding, Key Laboratory of Animal Genetics and Breeding of the Ministry of Agriculture, Beijing Key Laboratory for Animal Genetic Improvement, College of Animal Science and Technology, China Agricultural University, Beijing 100193, China; hanhongbing@cau.edu.cn

**Keywords:** *FOXL2*, granulosa cells, theca cells, hierarchical follicles, laying hen, leptin, ovary, prehierarchical follicles, *RSPO1*

## Abstract

**Simple Summary:**

In highly efficient laying hens, such as in commercial layer lines, the development of follicles is mainly characterized by an organized follicular hierarchy. Leptin has been implicated in the modulation of female reproduction in vertebrate animals. *Forkhead box L2* (*FOXL2*) and *R-spondin1 (RSPO1)* have also been implicated in the regulation of ovarian functions and the development of follicles. In this study, using a laying hen model, we observed abundant mRNA expression of *FOXL2 and RSPO1* in small (prehierarchical) and large (hierarchical) follicles, respectively. *FOXL2* mRNA expression was stable in granulosa cells harvested from 3–5 mm to F4 follicles, and exhibited a significantly higher expression in large hierarchical follicles. However, theca cells exhibited a significantly higher mRNA expression of *RSPO1* in F4 to F1 follicles. In subsequent experiment, we observed that 100 ng/mL leptin significantly modulated *FOXL2* and *RSPO1* expression in cultured granulosa cells harvested from large hierarchical and small prehierarchical follicles. These findings reasonably suggest that *FOXL2* and *RSPO1* genes may have a role in modulating the ovarian mechanisms, possibly affecting follicle growth and selection in laying hens. In addition, we demonstrated that leptin administration to granulosa cells in vitro modulated *FOXL2* and *RSPO1* expression, suggesting an implication of leptin in the follicular development and steroidogenesis in laying hens. However, further focused studies are warranted to improve our understanding of the exact roles played by these genes in follicle development and selection in laying hens.

**Abstract:**

In this study, using a laying hen model, we determined the expression of *FOXL2* and *RSPO1* in different central and peripheral tissue and ovarian follicles at different stages of development. At the same time, mRNA expression of both genes in granulosa and theca cells harvested from follicles at different stages of folliculogenesis was also evaluated. Finally, we assessed the effect of leptin treatment on expression of *FOXL2* and *RSPO1* in in vitro cultured granulosa cells harvested from 1–5 mm to F3–F1 follicles. Our RT-qPCR results revealed that a comparatively higher expression of *FOXL2* and *RSPO1* was observed in ovary, hypothalamus, and pituitary. Abundant mRNA expression of *FOXL2* was observed in small prehierarchical follicles (1–1.9 and 2–2.9 mm follicles; *p* < 0.05), whereas mRNA expression of *RSPO1* showed an increasing trend in large hierarchical follicles (F5–F1), and its abundant expression was observed in post-ovulatory follicles. *FOXL2* mRNA expression was stable in granulosa cells harvested from 3–5 mm to F4 follicles, and exhibited a significantly higher expression in large hierarchical follicles. Conversely, relatively low mRNA expression of *FOXL2* was observed in theca cells. *RSPO1* mRNA expression was relatively lower in granulosa cells; however, theca cells exhibited a significantly higher mRNA expression of *RSPO1* in F4 to F1 follicles. In the next experiment, we treated the in vitro cultured granulosa cells with different concentrations (1, 10, 100, and 1000 ng/mL) of exogenous leptin. Compared to the control group, a significant increase in the expression of *FOXL2* was observed in groups treated with 1, 10, and 100 ng/mL leptin, whereas expression of *RSPO1* was increased in all leptin-treated groups. When treated with 100 ng/mL leptin, *FOXL2* and *RSPO1* expression was upregulated in cultured granulosa cells harvested from both large hierarchical (F3–F1) and small prehierarchical follicles (1–5 mm). Based on these findings and evidence from mainstream literature, we envisage that *FOXL2* and *RSPO1* genes (in connection with hypothalamic-hypophysis axis) and leptin (via modulation of *FOXL2* and *RSPO1* expression) might have significant physiological roles, at least in part, in modulating the ovarian mechanisms, such as follicle development, selection, and steroidogenesis in laying hens.

## 1. Introduction

The reproductive strategy of avian species is unique compared to mammals, in that they usually produce a clutch of eggs that is dependent upon the maintenance of a small cohort of viable (undifferentiated) prehierarchical follicles [1,2]. In highly efficient laying hens, the development of follicles is mainly characterized by an organized follicular hierarchy [1,3]. Approximately, on a day-to-day basis, a single follicle is recruited from the pool, which undergoes a rapid growth phase and differentiation before ovulation [1]. These growing ovarian follicles are usually classified on the basis of their size (for example: 3–5 or 6–9 mm) or their color (as large white follicles or small yellow follicles). Classically, the ovarian follicles are mainly categorized as prehierarchical (≤9 mm in diameter) and hierarchical (>9 mm in diameter; designated as F5–F1: F5 < F4 < F3 < F2 < F1) follicles [4].

A few signaling pathways have been implicated in the process of follicle recruitment in domestic laying hens [1,5,6,7]; however, the exact underlying molecular mechanisms are still unclear. It has been reported that surrounding somatic cells have a well-coordinated interplay with the oocytes, and this interaction is believed to promote the process of follicle recruitment from the available pool [3]. The stage of development of ovarian follicles is associated with the initiation of steroidogenic competence in granulosa cell layers. Apparently, this process seems to be complex and highly coordinated, involving a number of divergent biological effects on the maturation of oocytes and differentiation and proliferation of granulosa and theca cells within the ovarian follicles [8]. In addition, the follicle-stimulating hormone (FSH) has been implicated in facilitating the recruitment of follicles [6,7,9,10,11,12]. Similarly, follicle stimulating hormone receptors (*FSHR*) are reportedly expressed on the granulosa layer of developing follicles, and mRNA expression of these receptors (*FSHR*) changes with progressing follicular maturation [3,6,13].

*Forkhead box L2* (*FOXL2*) is a highly conserved gene and encodes a forkhead transcription factor that is implicated in the development of gonads—in particular, granulosa cell differentiation, follicle development, and maintenance in many vertebrate species, including chickens [14,15,16,17,18]. Chicken *FOXL2* is located on chromosome 9, and its DNA-binding sequence depicts a high level of similarity to the mammalian counterpart. However, studies focusing on expression and functional implication of *FOXL2* in avian follicle growth and development are still sparse [16,17,18,19,20]. Previously, it was reported that *FOXL2* was abundantly expressed in granulosa cell layers in chicken ovarian tissue [21]. It was also demonstrated that FOXL2 protein is critical for early regulation of ovarian development in avian species and might have an implication in regulation of aromatase transcription [21]. Besides, in a recent transcriptomic study [16], it was suggested that *FOXL2* might play different stage-specific functional roles in development of chicken granulosa cells [16].

In mammalian species, *R-spondin1* (*RSPO1*), an activator of the WNT/β-catenin signaling pathway, is located upstream of the female sex determination pathway [22,23,24]. However, in non-mammalian species, the potential roles of *RSPO1* in ovarian differentiation remain largely unclear [25,26,27]. In one previous study on a chicken embryo model, it was demonstrated that *RSPO1* expression was elevated in females at the time of ovarian differentiation, concurring with female-specific activation of the *FOXL2* and estrogen synthesis [28]. Moreover, inhibition of estrogen synthesis with a specific aromatase inhibitor results in a reduced *RSPO1* expression in chickens, suggesting that RSPO1 is influenced by estrogen [28].

Leptin, a member of the type I helical cytokine family, has been implicated in many vital biological functions, including reproduction [29,30,31,32]. As with mammals, cloning of the leptin receptor in chickens and its subsequent expression in organs, such as in hypothalamus, pituitary, and ovaries, has demonstrated that leptin might be implicated in modulation of reproduction in avian species by acting both centrally (hypothalamic–hypophysis axis) and peripherally (ovaries) [33,34,35,36]. Even though there is a slim evidence that leptin produces its potential effects by centrally acting on the neuroendocrine axis, our understanding of the putative mechanisms implicated in modulation of leptin signaling in avian ovaries remain largely incomplete [37]. 

In the present study, using a laying hen model, we conducted an initial preliminary experiment to explore the expression pattern of *FOXL2* and *RSPO1* in different central and peripheral tissues, including ovary, hypothalamus, and pituitary tissue. Meanwhile, we evaluated the expression patterns of *FOXL2* and *RSPO1* in ovarian follicles (different diameter bands) and granulosa and theca cells harvested from follicles at different stages of development (i.e., prehierarchical and hierarchical follicles). Subsequently, we studied the potential implication of exogenous leptin in modulating the expression of *FOXL2* and *RSPO1* in granulosa cells cultured in vitro (at different stages of follicle development).

## 2. Materials and Methods 

### 2.1. Ethics Statement, Experimental Birds, and Specimen Collection

The protocols for all animal experiments were approved by the Animal Welfare Committee of Sichuan Agricultural University, Chengdu, China (Ethical approval date/code: AEWC2016, January 6, 2016). All procedures strictly conformed to the Guide for the Care and Use of Agricultural Animals in Research and Teaching.

We randomly selected 18 laying Lohmann pink hens (15 weeks old) from a flock of 1500 hens (hatched on the same day and grown in the experimental farm at Sichuan Agricultural University, Chengdu, China). All hens were kept in standard housing conditions and offered ad libitum access to feed and water. The laying sequence was carefully monitored (in 18 selected hens) before the animals were utilized in subsequent experiments. For specimen collection, birds were slaughtered by a humane method, preventing discomfort. Immediately after slaughter, the abdominal cavity was exposed through ventral midline incision and tissue (kidney, pituitary, hypothalamus, oviduct, ovary, muscle, brain, lung, heart, and liver) sample collection was performed. 

As for our experiments on ovarian follicles at different stages of development, we collected and sorted the ovarian follicles in the following order: prehierarchical follicles (1–1.9, 2–2.9, 3–3.9, 4–4.9, 5–5.9, 6–6.9, 7–7.9, and 8–9 mm diameter), hierarchical follicles (F1–F5: F1 > F2 > F3 > F4 > F5), and post-ovulatory follicles (POF). In addition, for *FOXL2* and *RSPO1* mRNA expression analysis, theca and granulosa cells from 3–5 and 6–9 mm prehierarchical follicles and F5–F1 hierarchical follicles were collected concurrently. The theca and granulosa cells were separated as described previously [37,38]. All samples were collected in three replicates, snap frozen in liquid nitrogen, and stored at −80 °C until total RNA extraction.

### 2.2. Granulosa Cell Culture and Leptin Treatment

Granulosa cells collected from prehierarchical and hierarchical follicles were washed with phosphate-buffered saline (pH 7.4) and digested with 0.1% (*w*/*v*) Type II collagenase (Sigma, St Louis, USA) at 38.5 °C for 6 min with gentle agitation in a flask. Cell viability was assessed by the Trypan blue dye exclusion test. The cells were diluted with the culture media to a concentration of 5 × 10^5^/mL and then seeded onto 6-well culture plates and incubated at 38.5 °C under 5% CO_2_ in humidified air to attain a desirable confluence. The media consisted of Dulbecco’s modified Eagle’s medium/nutrient mixture containing 3% fetal bovine serum (Sigma, St Louis, USA). 

To assess the influence of exogenous leptin treatment on expression of *FOXL2* and *RSPO1* in granulosa cells, after two days of preculture, the culture medium was replaced by a fresh medium with recombinant (mouse-like) leptin (Pro Spec-Tany Techno Gene Ltd., Rehovot, Israel) at different concentrations: 0, 1, 10, 100, and 1000 ng/mL. Then, after 24 h of culture, total RNA was extracted from granulosa cells in each group. Each group consisted of three replicates, and the same treatment was repeated in triplicate. The concentration of exogenous leptin (i.e., 0, 1, 10, and 100 ng/mL) used in this study was adopted from two previous reports [37,39]. It is important to note that the recombinant leptin used in this study was prepared from a previously known (erroneous) sequence, which is almost identical to the mouse leptin and different from the newly identified [40,41] genuine chicken leptin (less than 30% identity of the amino acid sequence).

### 2.3. RNA Extraction and cDNA Synthesis

Total RNA was extracted from all the samples using RNAiso Plus (Takara, Dalian, China) according to the manufacturer’s guidelines. The RNA integrity was evaluated by visualization of the 28S/18S rRNA ratio via 1.5% agarose gel electrophoresis. Then, the total RNA sample was treated with the gDNA Eraser Kit system (in a reaction containing 2 μL 5 × gDNA Eraser Buffer, 1 μL gDNA Eraser, 0.8 μL total RNA, and 6.2 μL H_2_O) for five minutes to remove the genomic DNA. The concentration of RNA was measured using a Beckman DU-640 nucleic acid/protein concentration spectrophotometer (Beckman, USA). The cDNA was obtained using a cDNA synthesis kit (PrimeScript^®^, Takara, Dalian, China), as per the manufacturer’s guidelines, using 1μg of total RNA as a template. The reverse transcription step was carried out in triplicate and the total RNA concentration was the same in every sample [42].

### 2.4. Quantitative Reverse Transcription PCR (RT-qPCR)

For mRNA expression analysis, we performed RT-qPCR using a CFX 96TM Real-Time PCR Detection System (Bio-Rad, USA), as described previously [4] with some modifications. Briefly, we used a 15 μL reaction mixture containing 1.5 μL of cDNA template, 6.5 μL of 2 × SYBR Premix Ex Taq II (Takara, Dalian, China), and 0.4 μL each of the forward and reverse primers. The PCR reaction conditions were as follows: (1) an initial denaturation at 95 °C for 30 s, followed by (2) 40 cycles of 95 °C for 5 s and (3) primer-specific annealing temperature for 30 s (see Table 1). Glyceraldehyde 3-phosphate dehydrogenase (*GAPDH*) and beta actin (*ACTB*) were used as reference genes [4,43]. For quality control and threshold cycle (Ct) calibration, we included a no template control (nuclease-free water instead of cDNA) and a negative control (without reverse transcriptase) in all technical replicates of PCR assays, as per our laboratory’s protocol. To validate the specificities of each target-specific primer pair, a melting curve analysis was performed as described previously [44]. Only one product of desired size was identified, and a single smooth peak was observed for each primer in melt curve analyses. Amplification (PCR) efficiencies (determined by 10-fold serial dilutions of cDNA, assayed in triplicates) of all the genes of interest and the internal reference genes were similar and closer to 100%, allowing the use of the 2^−ΔΔCt^ (Livak) method [45] to calculate the relative gene expression levels. In all experiments, each sample was run in triplicate. The details of the primers used in this study are shown in Table 1.

### 2.5. Statistical Analyses

**Figure 1:**
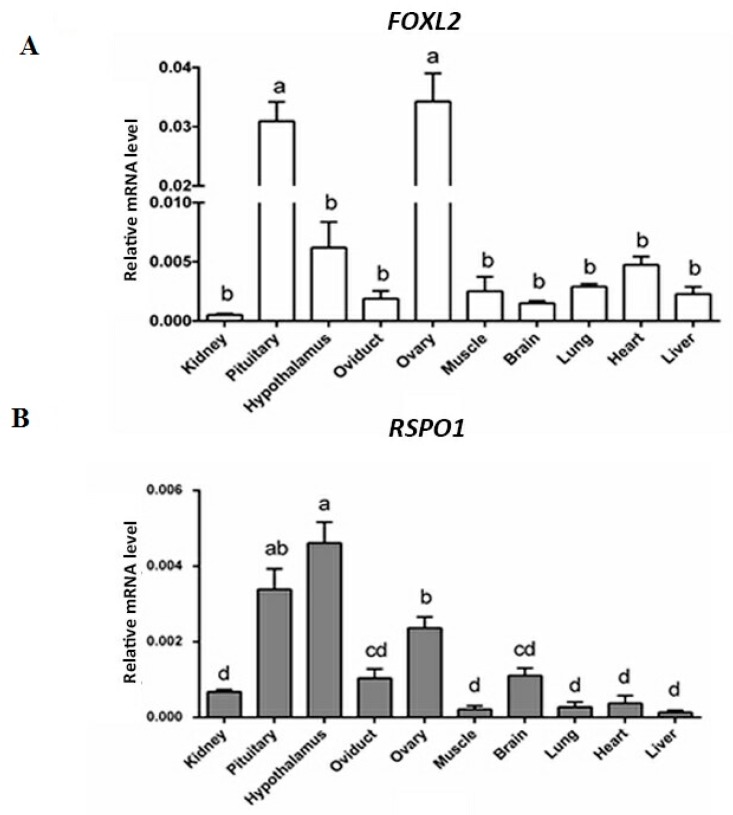
The mRNA expression of *FOXL2* and *RSPO1* in different tissues of laying hens. (**A**) *FOXL2* mRNA expression relative to *GAPDH* and *ACTB* mRNA. (**B**) *RSPO1* mRNA expression relative to *GAPDH* and *ACTB*. Values represent mean ± SEM, and bars with different superscripts are significantly different (*p* < 0.05). Data were analyzed using one-way ANOVA followed by post-hoc Duncan’s test.

**Figure 2:**
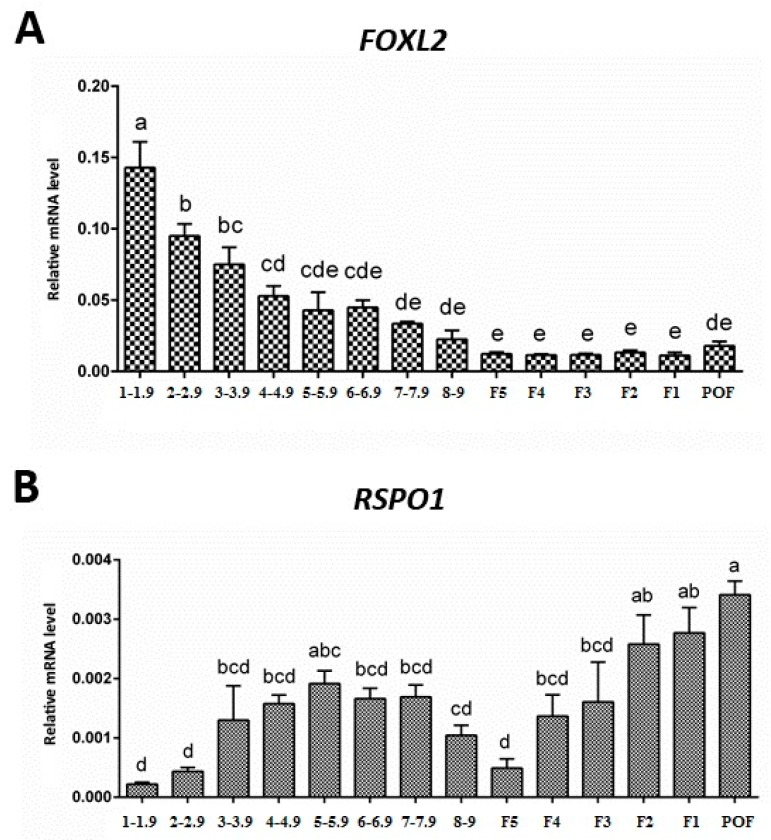
Expression of *FOXL2* (A) and *RSPO1* (B) mRNA in hen ovarian follicles at different stages of development. The mRNA expression levels are relative to *GAPDH* and *ACTB*. Bars (mean ± SEM) with different letters are significantly different (*p* < 0.05). Data were analyzed using one-way ANOVA followed by post-hoc Duncan’s test. Numbers in the x-axis (i.e., 1–1.9, 2–2.9, 3–3.9, 4–4.9, 5–5.9, 6–6.9, 7–7.9, and 8–9) show prehierarchical follicles grouped according to diameter (mm): 1–9 mm, prehierarchical follicles; F5 to F1, hierarchical follicles; POF, post-ovulatory follicles.

**Figure 3:**
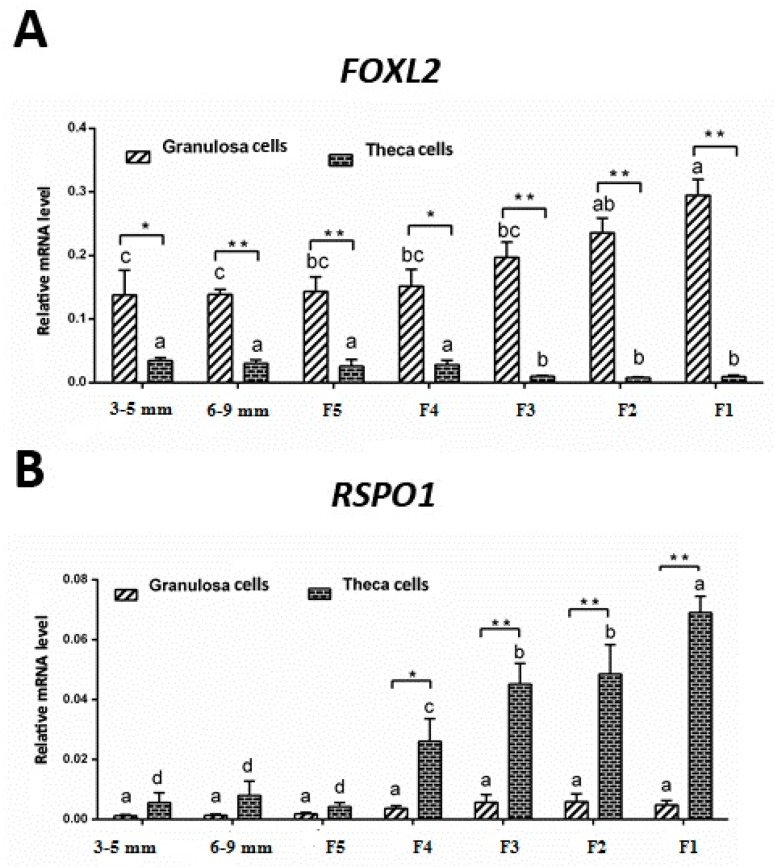
The mRNA expression of *FOXL2* (**A**) and *RSPO1* (**B**) in theca and granulosa cells harvested from follicles at different stages of development. Bars with different lowercase letters are significantly different between the same types of cells from follicles of different sizes (*p* < 0.05). Note: * and ** indicate significant differences between theca and granulosa cells in follicles of the same size *(p* < 0.05 and *p* < 0.01, respectively). Data were analyzed using one-way ANOVA followed by post-hoc Duncan’s test. The mRNA expression levels are relative to *GAPDH* and *ACTB*.

**Figure 4:**
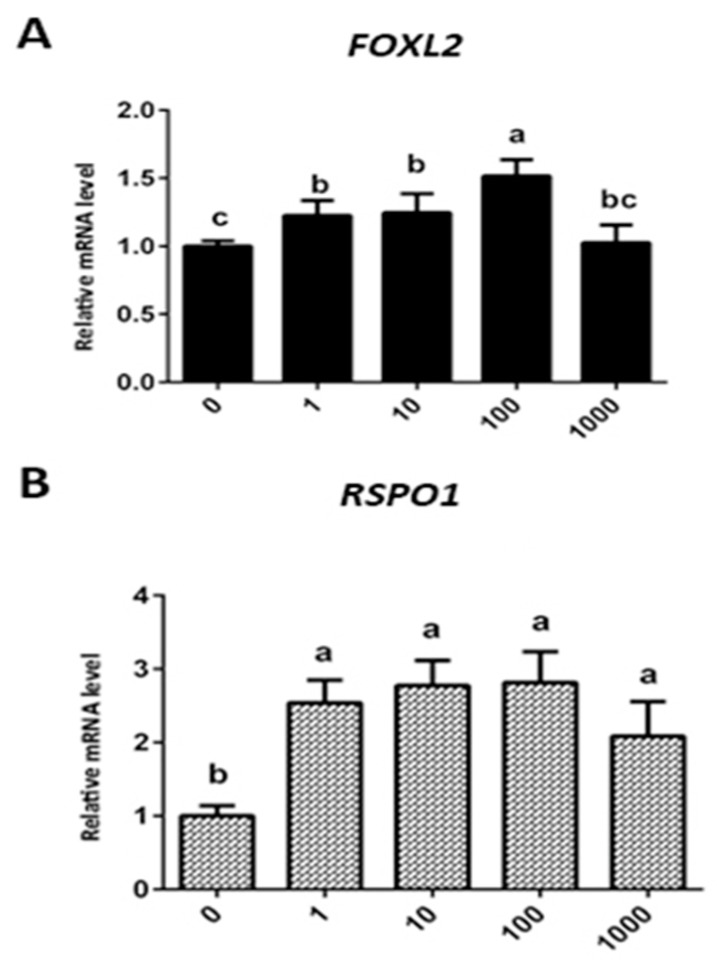
Effect of leptin (at different concentrations) on mRNA expression of *FOXL2* (**A**) and *RSPO1* (**B**) in cultured chicken granulosa cells. Values (mean ± SEM) with different lowercase letters are significantly different (*p* < 0.05). Data were analyzed using one-way ANOVA followed by post-hoc Duncan’s test. Values in the x-axis (0, 1, 10, 100, and 1000) represent different concentrations (ng/mL) of leptin. The mRNA expression levels are relative to *GAPDH* and *ACTB*.

**Figure 5:**
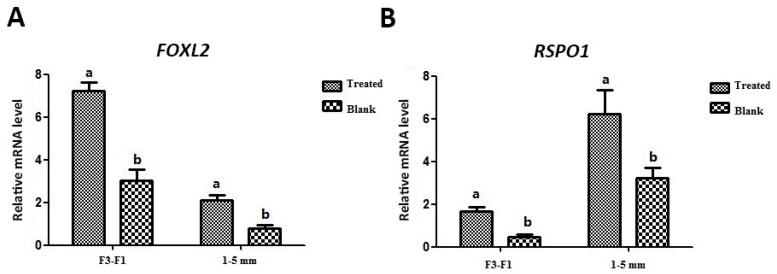
Effect of leptin (100 ng/mL) on mRNA expression of *FOXL2* (**A**) and *RSPO1* (**B**) in granulosa cells harvested from large hierarchical (F3–F1) and small prehierarchical follicles (1–5 mm). Values (mean ± SEM) with different lowercase letters within the same group are significantly different (*p* < 0.05). Data were analyzed using a Student’s *t*-test. The mRNA expression levels are relative to *GAPDH* and *ACTB*. Definitions: treated indicates granulosa cells treated with 100 ng/mL leptin; blank indicates control group without leptin addition.

All data shown in Figure 1, Figure 2, Figure 3 and Figure 4 were analyzed using one-way analysis of variance (ANOVA), followed by a post-hoc Duncan’s test (SAS 9.4; SAS Institute, Cary, USA). Data on the effect of leptin (100 ng/mL; Figure 5) on mRNA expression of *FOXL2* and *RSPO1* in granulosa cells harvested from F3–F1 and 1–5 mm were analyzed using a Student’s *t*-test. All data are presented as mean ± standard error of the mean (SEM) of at least three independent replicates. A *p* value < 0.05 was considered to be statistically significant.

## 3. Results

### 3.1. mRNA Expression of FOXL2 and RSPO1 in Different Central and Peripherial Tissues of Laying Hens

We used RT-qPCR to analyze the mRNA expression of *FOXL2* and *RSPO1* in different tissues of laying hens. Detailed results are depicted in Figure 1. Briefly, the mRNA expression of both genes showed significantly higher levels in pituitary, hypothalamus, and ovary of laying hens. As for the *FOXL2*, a significantly higher (*p* < 0.05) mRNA expression was observed in ovaries and pituitary (Figure 1A). Conversely, the mRNA expression of *RSPO1* was significantly higher in the hypothalamus, followed by pituitary and ovary (Figure 1B).

### 3.2. Expression of FOXL2 and RSPO1 in Ovarian Follicles at Different Stages of Development

The mRNA expression levels of *FOXL2* and *RSPO1* were determined in ovarian follicles at different stages of development in laying hens. Our RT-qPCR results revealed that *FOXL2* and *RSPO1* were expressed in ovarian follicles at all stages of development (Figure 2). The abundant mRNA expression of *FOXL2* was observed in small prehierarchical follicles, in particular in follicles ranging 1–1.9 and 2–2.9 mm (*p* < 0.05). Notably, a decreasing trend was observed in *FOXL2* mRNA expression in prehierarchical follicles at different developmental stages (i.e., from 1–1.9 mm to 8–9 mm follicles (Figure 2)). However, a stable low mRNA expression was observed in hierarchical follicles from F5 to F1 (Figure 2). In contrast to *FOXL2*, we observed a significantly low (*p* < 0.05) mRNA expression of *RSPO1* in small prehierarchical (1–1.9 mm) follicles. However, the expression pattern showed an increasing trend from 2–2.9 to 5–5.9 mm follicles and again decreased in 6–6.9 mm to F5 follicles. Interestingly, the mRNA expression of *RSPO1* again showed an increasing trend in large hierarchical follicles (F5–F1) and its abundant expression (*p* < 0.05) was observed in POF (Figure 2).

### 3.3. mRNA Expression of FOXL2 and RSPO1 in Theca and Granulosa Cells of Ovarian Follicles

In the next experiment, we evaluated the expression patterns of *FOXL2* and *RSPO1* in granulosa cells and theca cells obtained from follicles at different stages of development. Our results revealed that mRNA expression of *FOXL2* was stable in granulosa cells harvested from 3–5 mm to F4 follicles, and exhibited a significantly higher (*p* < 0.05) expression in large hierarchical follicles. Conversely, relatively low levels of mRNA expression were observed in theca cells. However, *FOXL2* mRNA expression was relatively higher in theca cells from small follicles (3–5 mm, 6–9 mm, and F5–F4) compared to the large hierarchical follicles (F3 to F1). Moreover, in contrast to *FOXL2*, the mRNA expression of *RSPO1* was relatively lower and non-significant in granulosa cells obtained from follicles at different stages of development. On the other hand, after relatively lower expression levels in 3–5 mm to F5 follicles, theca cells exhibited a significantly higher (*p* < 0.05) mRNA expression level (in an increasing trend) of *RSPO1* in F4 to F1 follicles (Figure 3). Intriguingly, *FOXL2* mRNA expression levels in granulosa cells of F4 to F1 follicles were significantly higher (*p* < 0.05) compared to the theca cells, whereas the mRNA expression levels of *RSPO1* showed a reverse tendency, i.e., mRNA expression levels were significantly higher (*p* < 0.01) in theca cells of F4 to F1 follicles compared to the granulosa cells (Figure 3).

### 3.4. Effect of Exogenous Leptin Treatment on mRNA Expression of FOXL2 and RSPO1 in Granulosa Cells (In Vitro Cultured)

In order to assess the influence of exogenous leptin treatment on expression of *FOXL2* and *RSPO1*, we treated the in vitro cultured granulosa cells with different concentrations of exogenous leptin. Our results revealed that mRNA expression of both genes was upregulated following leptin treatment at all concentrations. Of note, compared to the control group, a significant increase (*p* < 0.05) in expression of *FOXL2* was observed in groups treated with 1, 10, and 100 ng/mL leptin, whereas a significant increase (*p* < 0.05) in expression of *RSPO1* was observed in all leptin-treated (1, 10, 100, and 1000 ng/mL) groups. 

However, it should be noted that abundant expression of both *FOXL2* and *RSPO1* was observed at the peak in the 100 ng/mL leptin-treated group, and relatively lower expression (compared to other treated groups) was observed in the 1000 ng/mL leptin-treated group (Figure 4). Therefore, bearing in mind these observations, we chose 100 ng/mL as the standard concentration for subsequent experiments, focusing on stage-dependent influence of leptin on expression of *FOXL2* and *RSPO1* in granulosa cells. To this effect, we treated in vitro cultured granulosa cells harvested from two groups of ovarian follicles separately, i.e., large hierarchical (F3–F1) and small prehierarchical follicles (1–5 mm). We observed that *FOXL2* and *RSPO1* were significantly (*p* < 0.05) upregulated (Figure 5) in granulosa cells in both groups of follicles (F3–F1 and 1–5 mm). 

## 4. Discussion

Even though the significance of cell-autonomous FOXL2 action to proper ovarian maturation and function has been demonstrated, the implication of pituitary FOXL2 in modulation of the reproductive axis remains poorly understood [46]. It has been reported that FOXL2 functions at multiple levels of the hypothalamic–pituitary–gonadal (HPG) axis. Justice and colleagues demonstrated that FOXL2 is required for the appropriate expression of FSH by pituitary gonadotropes and elicited the assumption that pituitary actions of FOXL2 contribute to the established function of this forkhead protein in the development of ovaries [46]. Similarly, it has been speculated that *RSPO1* could represent the key ovary-determining gene in human beings, and presumably all amniotic vertebrates, including chickens. It has been suggested that RSPO1 might act in connection with Wnt4 in the developing ovary [28]. Given that it engages the effector pathway of Wnt signaling, β-catenin, in other systems, it is possible that RSPO1 has a potential functional role in the development of ovaries by modulating β-catenin. It has been argued that β-catenin has two potential functions at the level of cell i.e., trans-activation of target genes and the formation of adherent type junctions, either or both of which might be essential for ovarian differentiation [24,28]. Bearing in mind foregoing observations, it reasonable to speculate that as observed in our study, the abundant expression of *FOXL2* and *RSPO1* in pituitary, hypothalamus, and ovary might be indicative of potential implication of these genes in modulating the hypothalamic–pituitary–gonadal axis in laying hens, thereby regulating the growth, development, and selection of ovarian follicles. Nevertheless, further functional and well-powered mechanistic studies will be of great value to support this caveat. 

It is well-established that development of chicken ovarian follicles is a complex and highly regulated process in which several endocrine, paracrine, and autocrine factors within the follicles are implicated in a spatiotemporal manner to modulate and coordinate the growth and development of the oocyte, granulosa, and theca cells [17]. In chicken ovarian development, both follicular viability and differentiation following follicle selection are dependent on FSH stimulation and the expression of FSH receptors in granulosa cells [6]. In one earlier landmark study on chicken *FOXL2*, Govoroun and colleagues [21] reported an abundant expression of *FOXL2* in granulosa cells in mature hen ovaries. Besides, Qin and colleagues reported a new SNP (single-nucleotide polymorphism) in *FOXL2* affecting egg production and egg weight in Chinese Dagu hens [18].

Lately, FOXL2 has also been implicated in a bidirectional modulating role associated with the intracellular FSH receptor transcription and granulosa cell proliferation through an autocrine regulatory mechanism in a positive or negative manner in cooperation with activin A or GDF9, and follistatin in the chicken follicle development [17]. Similarly, our data suggest that *FOXL2* mRNA expression levels were abundant in 1–1.9 and 2–2.9 mm follicles, and decreased with the development of prehierarchical follicles (1–9 mm), with stable low expression levels in the hierarchical follicles (F5–F1), indicating that *FOXL2* might play a role in follicle selection and the development of prehierarchical follicles. These observations strongly indicate that FOXL2 factor performs an essential role by modulating the follicle development and granulosa cell proliferation and differentiation in prehierarchical follicles in a well-controlled and coordinated manner in hens [17].

Moreover, in the past it has been suggested the *FOXL2* might regulate the ovarian steroidogenesis and normal ovarian follicle development. This role could either be accomplished by binding to steroidogenic factor 1 (SF-1) or acting as a co-regulator of nuclear receptors [47]. However, in significant contrast to this notion, recently it has been suggested that interplay between FOXL2 and CYP19A1 in hen ovaries might be distinct from that in mammalian species [48]. However, in a very recent report, Zhang and colleagues demonstrated that *FOXL2* might regulate the ovarian follicle development and granulosa cell function in mature chicken ovaries [49]. Interestingly, similar to our findings regarding the abundant expression of *FOXL2* in granulosa cells harvested from prehierarchical and hierarchal follicles, recently in a transcriptome analysis of chicken prehierarchical and preovulatory granulosa cells, Wang and colleagues observed that *FOXL2* was abundantly expressed in granulosa cells at all stages of follicle development, and expression levels increased after follicle selection, indicating that *FOXL2* may have an essential functional role in chicken granulosa cell differentiation and follicle development [16]. These researchers further speculated that *FOXL2* might be implicated in chicken granulosa cell growth and differentiation via activation of the PI3K/AKT pathway through the induction of cytokine expression [16]. There is a possibility that *FOXL2* might play different roles at different stages of granulosa cell development in hens, i.e., promoting follicle selection in prehierarchical granulosa cells and repressing ovulation in preovulatory granulosa cells [16]. Therefore, the window is definitely open to elucidate the potential implication of *FOXL2* in regulating the ovarian follicle development and steroidogenesis in laying hens.

Intriguingly, the expression pattern of *RSPO1* showed a reverse tendency compared to *FOXL2*; its expression was significantly lower in small prehierarchical follicles, and increased consistently in large hierarchal follicles (F5–F1). It is worth mentioning that due to the lack of evidence on this aspect, we are unable to relate our observations to that of others. However, in one of the very sparse studies on expression of chicken *RSPO1,* Smith and colleagues comprehensively demonstrated that *RSPO1* expression becomes elevated in females at the time of ovarian differentiation, concurring with female-specific activation of the *FOXL2* gene and synthesis of estrogen [28]. Moreover, these authors further reported that inhibition of estrogen synthesis with a specific aromatase inhibitor leads to reduced *RSPO1* expression in chicken, suggesting that *RSPO1* is potentially influenced by estrogen [28]. In this study, we observed that *RSPO1* was abundantly expressed in theca cells of ovarian follicles in laying hens, in particular at later stages of development (i.e., in theca cells harvested from large hierarchal follicles (F4–F1)). 

Previously, it has been demonstrated that *RSPO1* is abundantly expressed in somatic cells of chicken ovaries, however, the protein was found to be localized to the cell surface and cytoplasm of both germ and somatic cells [28]. It is reiterated that these observations suggest that RSPO1 might have an important role in follicle development. It has also been speculated that *RSPO1* may lie downstream of aromatase in the avian ovarian pathway. However, a previous study on aromatase inhibition suggested that estrogen synthesis is needed to maintain the expression of *RSPO1*, perhaps by sustaining the cortical prefollicular cell population. Nevertheless, further studies are awaited to elucidate the putative regulatory mechanisms by which *RSPO1* expression is modulated in follicles at different stages of development, and to understand its mechanism of action, which is possibly mediated by an interplay with WNT signaling and stabilization of β-catenin [22,23,28]. In mammalian models, although it has been demonstrated that *FOXL2* and *RSPO1* seem to act in a complimentary manner via a direct or indirect interaction involving the WNT signaling pathway (involving *Wnt4*), during ovarian differentiation [50], the precise mechanistic basis for these complex interactions still remains to be explored. 

One of the intriguing observations of the present investigation was the portraying of exogenous leptin-modulated mRNA expression pattern (in a dose-dependent manner) of *FOXL2* and *RSPO1* in ovarian follicles and granulosa cells harvested from chicken ovarian follicles at different stages of development. The results of our study demonstrated that leptin treatment abundantly upregulated the expression of *FOXL2* and *RSPO1* at different doses, except for the high dose 1000 ng/mL group, in which the change in *FOXL2* expression was non-significant. However, the optimal upregulation of *FOXL2* and *RSPO1* expression (both in follicles and cultured granulosa cells) was observed in the 100 ng/mL group compared to other treatment groups. However, it is important to note that there is a possibility that in vitro conditions and exogenous recombinant leptin treatment may not necessarily mirror the in vivo conditions and endogenous genuine leptin effects. Therefore, further focused studies are required to reinforce these findings.

It is worth mentioning that previously leptin has been implicated in regulating various reproductive functions in females by acting centrally on the hypothalamic–hypophysis axis and peripherally at the level of ovary [29,44]. However, concrete evidence regarding its implication in avian models is still sparse, and only a few reports have reported leptin modulation of ovarian function in birds to date [35,36,37,39,51,52]. Although we were unable to find studies focusing on the leptin regulation of *FOXL2* and *RSPO1* expression patterns in ovarian follicles and in vitro cultured granulosa cells, some of the previous reports have, however, demonstrated that leptin might modulate the proliferation, apoptosis, and secretory activity of cultured chicken ovarian cells [36,37,44]. Using a goose model, Hu and colleagues recently reported that *LEPR* gene expression was abundantly observed in granulosa and theca cells harvested from follicles of 4–8 mm diameter. However, in granulosa cells, this expression gradually decreased as the follicle development advanced (F5 and F3) [37]. These authors further reported that cultured goose granulosa cells showed significantly increased levels of estradiol when cultured in vitro. In addition, leptin treatment significantly upregulated the expression of sterol/steroid biosynthesis-related genes, such as sterol regulatory element binding protein 1 (*SREBP1*) and cytochrome P450 14α-sterol demethylases (*CYP51*) in goose granulosa cells. Therefore, it is reasonable to infer that leptin could modulate the synthesis of steroid hormones via interplay with its receptors in avian ovarian cells [37]. Besides, previously it has been demonstrated that leptin could directly modulate the basic chicken ovarian functions, such as inhibition of cytoplasmic apoptosis and proliferation (S-phase of mitosis), regulation of secretory activity (release of steroid hormones), and expression of MAPK, PKA, and CDC2, which might be potential intracellular mediators of leptin action in avian species [39]. Similarly, Song et al. also demonstrated that leptin treatment, at moderate doses, ameliorates the function of regressed ovaries in ducks. Leptin also promoted the recovery of yellow hierarchical follicles and increased the plasma estradiol (E_2_) level, and increased the mRNA expression of *FSHR*, luteinizing hormone receptor (*LHR*), and estrogen receptor (*ER*) in duck ovaries [51]. Interestingly, it has also been suggested that leptin might have an important physiological role in modulating the sexual maturation in laying hens, and that its action is potentially linked with ameliorating the onset of apoptosis in ovarian cells and improved folliculogenesis [36]. However, it is largely elusive whether leptin stimulates the development of ovarian follicles by acting solely at the central level or also at the peripheral level. It also remains unknown whether leptin is a critical, permissive, or facilitator factor in its regulation of puberty in hens [36]. Nevertheless, in one recent report [44], Wen and colleagues, using goose granulosa cells (harvested from ovarian F1–F3 preovulatory follicles) as a model, demonstrated that leptin treatment increased the expression of some of the key biomarkers, such as phosphatidylinositol 3-kinase (PI3K), Akt1, Akt2, Raptor, mammalian target of rapamycin (mTOR), S6 kinase (S6K), and phosphorylated S6 kinase (p-S6K). These findings provide a reasonable evidence that leptin might play proliferative and anti-apoptotic roles in avian granulosa cells through the PI3K/Akt/mTOR signaling pathway via interaction with its receptor [44]. These findings also support the notion regarding the peripheral actions of leptin at the level of ovaries in birds.

It also pertinent to note that recent findings by Seroussi and colleagues [40] have ended the long-standing controversy regarding the existence of the true chicken leptin ortholog. Finally, genuine chicken leptin was found [40] and validated recently by genomic mapping with five syntenic genes [41]. In addition, these findings have also reshuffled the hypothesis pertaining to the potential role and mode of action of leptin in avian species [40]. Moreover, recent findings have revealed that genuine avian leptin is strikingly different from mammals orthologs, as it is expressed at very low levels (or not at all) in adipose tissues [53]. The mRNA expression profiling of *LEP* in many avian species, including chickens, has demonstrated its expression in various central (hypothalamus and pituitary) and peripheral (gonads) tissues [40,53]. In addition, mRNA expression levels of avian *LEPR* are also tightly correlated to that of *LEP* mRNA, intriguingly suggesting a paracrine or autocrine mode of action in avian species [40,53]. 

Therefore, in view of the foregoing evidence, it is necessary to acknowledge that given the fact that the exogenous leptin used in this study was prepared using a previously known erroneous sequence of *LEP*, it is possible that the in vitro effects of exogenous leptin treatment on mRNA expression of *FOXL2* and *RSPO1* genes in granulosa cells as observed in the present study could be peculiar to the recombinant mouse-like leptin, and that the genuine endogenous chicken leptin may function differently at the level of the ovaries. However, it also important to mention that recombinant (mouse-like) leptin is also expected to bind and activate the chicken leptin receptor (*cLEPR*). This notion is based on the demonstration of binding and activation of a variety of leptins and *LEPRs* across species [54,55]. Lastly, based on these facts, it is highly recommended that the results of present study and those of other studies [35,36,37,39,44,51] using recombinant (mouse-like) leptins published before the discovery and validation of genuine chicken leptin [40,41] should be substantiated or revisited using a true ortholog of chicken leptin.

## 5. Conclusions

In the present study, using a laying hen model, we demonstrate that *FOXL2* and *RSPO1* genes were expressed in a stage-dependent pattern in ovarian follicles and granulosa and theca cells, and that the exogenous (mouse-like) leptin administration in in vitro cultured granulosa cells modulated *FOXL2* and *RSPO1* expression, suggesting a potential implication of leptin in the follicular development and selection in domestic hens.

In summary, bearing in mind the novel observations of our study and evidence from mainstream literature, we envisage that *FOXL2* and *RSPO1* genes, in connection with the hypothalamic–hypophysis axis, might have significant physiological roles, at least in part, in modulating the ovarian mechanisms, such as follicle growth, development, and selection in laying hens. Similarly, based on our in vitro experiment results, we also envisage that leptin, via modulation of *FOXL2* and *RSPO1* expression, might have implications in follicular growth, development, and steroidogenesis in laying hens. However, given the fact that the exogenous leptin used in the current study was prepared from a previously known erroneous sequence and not the newly discovered and validated genuine sequence [40,41], it is necessary to conduct further focused studies to comprehensively understand the potential roles and underlying molecular mechanisms through which the true ortholog of chicken leptin performs its (yet to be fully elucidated) functions in physiologically unique ovarian follicular biology in avian species.

## Figures and Tables

**Table 1:** Details of primer used in this study.

Primer Name	Primer Sequence (5′→3′)	Annealing Temperature (°C)	Accession Number	Product Length (bp)
*FOXL2*-F	CCTCAACGAGTGCTTCATCA	60	NM_001012612	299
*FOXL2*-R	ACATCTGGCAAGAGGCGTAG			
*RSPO1*-F	AAGGCTACTCTGCTGCCAAC	60	NM_001318444	295
*RSPO1*-R	CGATTTCTGTTCCCGTTTGT			
*GAPDH*-F	CTTTCCGTGTGCCAACCC	61	NM_204305.1	136
*GAPDH*-R	CATCAGCAGCAGCCTTCACTAC			
*ACTB*-F	TGGGTATGGAGTCCTGTGGT	60	L08165.1	160
*ACTB*-R	AGGGCTGTGATCTCCTTCTG			

Note: F, forward; R, reverse.
